# Communication of Intent to Do Harm Preceding Mass Public Shootings in the United States, 1966 to 2019

**DOI:** 10.1001/jamanetworkopen.2021.33073

**Published:** 2021-11-04

**Authors:** Jillian Peterson, Gina Erickson, Kyle Knapp, James Densley

**Affiliations:** 1 Department of Criminal Justice and Forensic Science, Hamline University, St Paul, Minnesota; 2 College of Criminology and Criminal Justice, Florida State University, Tallahassee; 3 School of Law Enforcement and Criminal Justice, Metropolitan State University, St Paul, Minnesota

## Abstract

**Importance:**

Understanding the motivation of a mass shooter’s intent to do harm can help practitioners and policy makers develop more effective intervention strategies.

**Objective:**

To examine the prevalence of communication of intent to do harm, known as leakage, in a sample of 170 mass public shooters from 1966 to 2019; the characteristics of perpetrators who do and do not leak their plans; and whether leakage is a form of fame-seeking behavior or a cry for help among individuals who are in crisis or suicidal.

**Design, Setting, and Participants:**

This cross-sectional study included perpetrators who killed 4 or more people in a public space from 1996 to 2019 and were included in a comprehensive database of US mass shootings. That database was built from August 2017 to December 2019, and analysis took place from January to May 2021.

**Main Outcomes and Measures:**

Leakage was identified and coded using publicly available records. Any communication to a third party of an intent to do harm prior to the shooting was coded as leakage. Logistic regression models were used to examine the risk factors associated with leakage. Models estimating leakage were examined to assess the 2 hypothesized pathways to leakage (the cry-for-help model and the fame-seeking model).

**Results:**

The 170 participants in this sample included 166 (97.7%) male perpetrators and 3 (2.3%) female perpetrators, with a mean (SD) age of 34 (12) years. Overall, 161 participants had known race and ethnicity: 11 (6.8%) Asian individuals, 35 (21.7%) Black individuals, 14 (8.7%) Latinx individuals, 7 (4.4%) Middle Eastern individuals, 3 (1.9%) Native American individuals, 89 (55.3%) White individuals, and 2 (1.2%) individuals with other race and ethnicity. Overall, 79 mass shooters (46.5%) leaked their plans. Of perpetrators who leaked their plans, 35 (44.3%) leaked specific plans about a mass shooting, and 44 (55.1%) leaked nonspecific plans about generalized violence. The study findings indicate that leakage was associated with receiving counseling (odds ratio, 7.0; 95% CI, 2.0-24.8) and suicidality (odds ratio, 3.7; 95% CI, 1.0-13.6), suggesting that leakage may best be characterized as a cry for help from perpetrators prior to their act.

**Conclusions and Relevance:**

In this study, nearly half of the perpetrators of mass shootings leaked their plans. Leakage was associated with receiving counseling and suicidality. Leakage is a critical moment for mental health intervention to prevent gun violence. Opportunities to report threats of violence need to be increased. Traditional threat assessment models focused on specific threats of violence may miss critical opportunities for intervention.

## Key Points

**Question:**

What factors are associated with a mass shooting perpetrator’s decision to communicate their intent to do harm?

**Findings:**

This cross-sectional study of 170 perpetrators found that nearly half leaked their plans, with 44.3% of them leaking specific plans about a mass shooting. Leakage was associated with receiving counseling and suicidality, which suggests it may be best characterized as a cry for help from perpetrators prior to their act.

**Meaning:**

These findings suggest that leakage is a critical moment for mental health intervention to prevent gun violence.

## Teaser

This cross-sectional study examines the prevalence of communication of intent to do harm, known as leakage, in a sample of 170 mass public shooters from 1966 to 2019, the characteristics of perpetrators who do and do not leak their plans, and whether leakage is a form of fame-seeking behavior or a cry for help among individuals who are in crisis or suicidal.

## Introduction

Mass public shootings, an event in which 4 or more people (not including the perpetrator) are killed with firearms in a public space, are a public health crisis.^1^ Mass shootings are statistically rare in comparison with other forms of gun violence, but because the perpetrators of mass shootings often have no relationship with their victims, every public space feels vulnerable (eg, schools, concerts, grocery stores, movie theaters, churches), and they require bespoke mitigation.^2^ One promising avenue for mass shooting prevention is threat assessment, which was born out of work by the US Secret Service after several high-profile attacks on public officials and figures.^3^

Threat assessment is designed to evaluate whether someone may engage in targeted violence, rather than other forms of violence and, consistent with a public health approach to violence reduction, is a deductive process focused on what a person’s behavior and communications (not characteristics) signal about their potential to do harm. A key concept in threat assessment is leakage, defined as communication to a third party of an intent to do harm to a target.^4^ Leakage is one of several types of warning behaviors that, when recognized and responded to, could prevent a mass shooting before it occurs.^5^

Understanding the phenomenon of leakage is critical for designing intervention strategies to prevent mass shootings. Few studies to date have examined leakage in the context of mass shootings. One study^6^ of the 15 deadliest shootings from 1998 to 2018 found that 87% of perpetrators leaked violent thoughts or intent and 80% leaked their interest in mass killing specifically. Another study of 30 adult mass murderers^7^ found some form of leakage in 67% of cases. The US Secret Service^8^ examined 37 school shootings, finding that 81% of the time, at least 1 other person knew about the shooting in advance. A study of 34 adolescent mass murderers^9^ found that 58% had made threatening statements and that leakage may be more common in younger perpetrators. The largest study to date^10^ of which we are aware examined 115 public mass shooters from 1990 to 2014 and identified leakage in 58% of cases. After exploring demographic differences between perpetrators who leaked and those who did not, the authors concluded there was no profile of a perpetrator who leaked their shooting in advance.^10^

Silver et al^10^ theorized that the motivation for perpetrator leakage may be attention seeking, intimidation, or excitement. Consistent with this theory, there is some evidence that perpetrators of mass shootings are narcissistic^11^ and committing a public mass shooting fulfills their quest for celebrity status.^12^ Media coverage of mass shootings is intense,^13^ and some mass shootings are a form of public performance, meant to draw attention to one’s grievance and anger with the world. Mass shootings related to fame seeking have been increasing in recent years,^14^ leading to higher casualty counts that make headlines. Fame-seeking perpetrators are often young White males targeting schools.^15^

On the other hand, perpetrators of mass shootings are often actively suicidal prior to their attack, and many take their own lives—or provoke law enforcement to do so, known as suicide by cop—during them.^16^ Warning signs often are different when an attacker has suicidal motives vs a desire for attention or fame.^17^ Publicly leaking plans may function as a cry for help when perpetrators become increasingly suicidal and hopeless. A 2018 study of mass killings over the last century^18^ found that 59% of 185 perpetrators had either been diagnosed with a serious mental illness or had demonstrated serious signs of a mental illness. A 2021 study of mental health factors in the Violence Project Database (the same data source for the 170 perpetrators analyzed in this study) found that 59% of perpetrators had a mental health history, defined as a history of counseling, psychiatric medication, psychiatric hospitalization, or prior diagnosis of mental illness.^19^

Analysis of leakage within the broader context of the lives of mass shooters is needed. The present study goes beyond previous analyses by studying a longer time frame and examining other variables associated with leakage in the perpetrators’ lives. When leakage is identified, traditional responses include various forms of punishment (ie, suspension or expulsion from school, termination from job) and even criminal charges for threats of violence.^20^ A punitive response may be an appropriate intervention if leakage is a form of attention and fame seeking. On the other hand, if leakage is a cry for help, indicating suicidality and hopelessness that may lead to violence, then criminal charges may only exacerbate the mental health crisis. In this model, suicide prevention and crisis intervention strategies may be a more effective public health response to leakage.

This study examines leakage in a sample of 170 perpetrators of mass shootings from 1966 to 2019, including the prevalence of leakage and characteristics of perpetrators who did and did not leak their plans. Three logistic regression models are used to explore leakage: (1) a baseline model of leakage; (2) the cry-for-help model, which examines the association between leakage and mental health factors; and (3) the fame-seeking model, which examines the association between leakage and fame-seeking factors. Each model serves as a different hypothesis for leakage among mass shooters, with unique and important public health implications for mass violence prevention.

## Methods

This cross-sectional study was deemed exempt by the Hamline University institutional review board and granted a waiver of informed consent because it only used publicly available records for coding. Data were derived from the largest, most comprehensive database of mass shootings in the United States. Funded by the National Institute of Justice, The Violence Project’s database of 168 public mass shootings with 4 or more victims killed from 1966 to 2019 includes 170 mass shooters coded on 166 independent variables for analysis.^21^ The full database was built from August 2017 to December 2019 and is publicly available alongside a detailed methodology and codebook, including definitions of each variable.^21^ This study followed the Strengthening the Reporting of Observational Studies in Epidemiology (STROBE) reporting guideline.

### Coding

To code the variables of interest, the research team drew on first-person accounts, such as the perpetrators’ diaries, manifestos, suicide notes, social media and blog posts, audio and video recordings, interview transcripts, and personal correspondence. Secondary sources, such as existing mass shooter databases, media coverage, documentaries, biographies, monographs, academic journal articles, court transcripts, police records, medical records, school records, and autopsy reports, were also consulted. Each cell in the database is linked to the source used for coding.

Ten coders were trained on the codebook and the definition of variables, and each case and variable were coded 4 separate times by at least 3 independent coders to ensure reliability before being checked again by a designated database manager, who had final document control. The entire process of building the database took 3 years, and in the final review, changes were made to 2.0% of cells.

Once the codebook was finalized and coders were trained in its use, the database was populated as follows. First, each mass shooter meeting the inclusion criteria (using the Congressional Research Service definition^22^) was investigated twice by 2 separate coders working independently from each other. The 2 resulting data sets were then merged and compared, and any discrepancies were flagged and reconciled by consensus of the principal investigators, who examined all sources, did their own fact-checking, and weighed the quality and quantity of the evidence, typically giving precedence to primary source material. The database was then divided among the original coders and independently checked again, ensuring that coders did not check their own work. The database manager conducted a full and final check of every cell, and the principal investigators answered any queries resulting from the final check before approving publication.

### Measures

#### Leakage

There were 4 leakage variables. They were coded as follows: any communication to a third party of an intent to do harm prior to the shooting (yes or no), how leakage occurred (in person, in writing, via telephone or text message, or via internet/social media), who leakage was made to (mental health professional, family, wife/girlfriend, police, coworker, peer, teacher, or other), and whether leakage was specific (threatened a shooting) or nonspecific (threatened violence more generally).

#### Demographic Characteristics

Each perpetrator was coded on the following demographic variables that were used in this study: gender, race (Asian, Black, Latinx, Middle Eastern, Native American, and White), age (<21 vs ≥21 years), region (Midwest, South, Northeast, or West), year of shooting, and location of the shooting (school, workplace, or other). Each of these variables was coded using publicly available data.

#### Cry for Help

The following mental health related variables were used in this model, coded (as binary yes vs no) based on the available sources: history of counseling, notably depressed mood, psychiatric medication use, and role of psychosis in the crime (no role vs any role). These codes were based on all publicly available sources and should not be interpreted as formal diagnoses. Perpetrators were also coded on suicidality (none, suicidal prior to the shooting, intended to die in the shooting). Missing data were interpreted as no for the purpose of this analysis.

#### Fame Seeking

The following variables related to fame seeking were used in this study, coded as binary yes vs no based on the available sources: social media use, high degree of planning (ie, collected weapons, studied other shootings, scouted different locations), performance element to the crime (ie, leaving a manifesto, wearing a costume), interest in past mass shootings (ie, previous research or writing), and fame-seeking motivation (based on the perpetrator’s declared motive and subsequent investigations). Social media was coded as yes or no, controlling for year. More information on each variable is available in the publicly available codebook.

### Statistical Analysis

Bivariate χ^2^ analyses were conducted with several factors known to be associated with leakage, including perpetrator age, perpetrator race, location of the shooting, suicidality, mental health factors, and shooting motivation. Logistic regression models in Stata version 17 (StataCorp) were used to explore the factors associated with leakage in the data. All models control for region (Northeast, South, Midwest, or West), year, location (school, workplace, or other), race other than White, suicidality, intention to die in the shooting, and age. Using the full sample of cases was important because mass shootings are rare events. There was not a significant difference between the percentage of leakers before and after 1999, the year of the Columbine shooting, which inspired many copycats.^23^ From 1966 to 1998, 30 of 69 perpetrators (43.5%) leaked plans; from 1999 to 2019, 49 of 101 shooters (48.5%) leaked plans. The inclusion of year effects helped account for changes in access to leakage methods (eg, social media) because leakage prior to 1999 may be different in type but not in intent (ie, a cry for help is a cry for help). Understanding leakage from earlier shooters could provide more important information, as each early leakage event was much more likely to be independent of others.

Data analysis took place from January to May 2021. We first examined a baseline model with these controls, followed by models with mental health factors and a model testing fame-seeking behavior. Missing data were less than 3% for any variable. To account for missingness we used multiple imputations with 20 imputations. All hypothesis tests were 2-sided, and statistical significance was set at *P* < .05.

## Results

### Descriptive Data

The prevalence of leakage was examined among the 170 cases for the years 1966 to 2019. The sample included 166 (97.7%) male perpetrators and 4 (2.3%) female perpetrators with a mean (SD) age of 34 (12) years. Overall, 161 participants had known race and ethnicity: 11 (6.8%) Asian individuals, 35 (21.7%) Black individuals, 14 (8.7%) Latinx individuals, 7 (4.4%) Middle Eastern individuals, 3 (1.9%) Native American individuals, 89 (55.3%) White individuals, and 2 (1.2%) individuals of other race or ethnicity. In this sample, 79 mass shooting perpetrators (46.5%) communicated intent to do harm to a target, ie, they leaked their plans (Table 1). Of perpetrators who leaked their plans, 35 (44.3%) leaked specific plans about a mass shooting and 44 (55.1%) leaked nonspecific plans about generalized violence. All perpetrators who leaked their plans were men, and White shooters leaked their plans more frequently than those from other racial and ethnic groups (48 of 89 [53.9%] vs 31 of 81 [38.3%]; *P* = .04).

**Table 1.** Leakage Among Mass Shooters in the United States, 1966-2019

Characteristic	Perpetrators, No. (%) (N = 170)
Leakage	
Yes	79 (46.5)
No	91 (53.5)
Mode of leakage^a^	
In person	52 (65.8)
Letter	3 (3.8)
Other writing	5 (6.3)
Telephone or text message	6 (12.7)
Online	10 (13.0)
Other	3 (3.8)
Who leaked to^a^	
Mental health professional	3 (3.8)
Immediate family	6 (7.6)
Wife or girlfriend	14 (17.7)
Police	2 (2.5)
Coworker or boss	15 (19.0)
Friend or neighbor	8 (10.1)
Classmate	7 (8.9)
Teacher or staff member	2 (2.5)
Bartender or waitress	5 (6.3)
Other	17 (21.5)
Type of leakage^a^	
Specific	35 (44.3)
Nonspecific	44 (55.7)

^a^ For mode of leakage, who leaked to, and type of leakage, the denominator was 79, ie, the perpetrators who leaked their plans.

### Bivariate Analysis

Perpetrators who leaked their plans were younger than those who did not (mean [SD] age, 30.8 [11.2] years vs 36.8 [12.4] years; *t* = 3.3; *P* = .001) and killed more people during the shooting than those who did not leak their plans (mean [SD] victims, 8.5 [7.5] vs 6.1 [6.0]; *t* = 2.3; *P* = .02). Mass shooters at kindergarten through 12th grade schools were significantly more likely to leak their plans (12 of 13 [92.3%]) than shooters at other locations (eg, college: 4 of 9 [44.4%]; *P* < .001). Of school shooters who leaked their plans, most (8 [61.5%]) did so to a peer, girlfriend, or acquaintance, and nearly all had a demonstrable interest in firearms (11 [84.6%]) and were suicidal (12 [92.3%]). Full descriptive information is shown in Table 2.

**Table 2.** Bivariate Results of Factors Associated With Leakage Among Perpetrators of Mass Shootings in the United States, 1966-2019

Characteristic	No. (N = 170)	Perpetrator, No. (%)		χ^2^ (df)	Missing cases, No. (% imputed)
		Did not leak (n = 91)	Leaked (n = 79)		
Age					
≥21 y	149	88 (59.1)	61 (40.9)	14.8 (1)^a^	NA
≤20 y	21	3 (14.3)	18 (85.7)		
Location					
K-12 school	13	1 (7.7)	12 (92.3)	19.9 (8)^b^	NA
College	9	5 (55.6)	4 (44.4)		
Government	6	4 (66.7)	2 (33.3)		
Place of worship	11	5 (45.5)	6 (54.6)		
Retail	29	14 (48.3)	15 (51.7)		
Restaurant/club	22	15 (68.2)	7 (31.8)		
Workplace	52	27 (51.9)	25 (48.1)		
Other	14	8 (57.1)	6 (42.9)		
Outdoors	14	12 (85.7)	2 (14.3)		
Race					
White	89	41 (46.1)	48 (53.9)	4.1 (1)^b^	NA
Other^c^	81	50 (61.7)	31 (38.3)		
Suicidality					
None	47	32 (68.1)	15 (31.9)	12.5 (2)^d^	NA
Suicidal before	53	18 (34.0)	35 (66.0)		
Intended to die	68	39 (57.4)	29 (42.7)		
Depression	51	20 (39.2)	31 (60.8)	6.00 (1)^b^	NA
Prior counseling	49	13 (26.5)	36 (73.5)	21.3 (1)^a^	2 (1.2)
Psychosis	51	34 (66.7)	17 (33.3)	5.2 (1)^b^	NA
Psychiatric medication	39	16 (41.0)	23 (59.0)	3.6 (1)	4 (2.4)
Social media use	41	14 (34.2)	27 (65.9)	7.7 (1)^d^	2 (1.2)
Planning	46	16 (34.8)	30 (65.2)	9.2 (1)^d^	1 (0.6)
Performance	17	5 (29.4)	12 (70.6)	4.4 (1)^b^	NA
Interest in past mass violence	40	13 (32.5)	27 (67.5)	9.7 (1)^d^	3 (1.8)
Fame-seeking motive	12	4 (33.3)	8 (66.7)	2.1 (1)	NA

Abbreviations: K-12, kindergarten through 12th grade; NA, not applicable.

^a^ *P* < .001.

^b^ *P* < .05.

^c^ Other race includes Asian, Black, Latinx, Middle Eastern, and Native American individuals.

^d^ *P* < .01.

### Multivariable Models

Multivariable models examining leakage were used to assess the 2 hypothesized pathways to leakage (the cry-for-help model and the fame-seeking model). Results are shown in Table 3.

**Table 3.** Multivariable Results^a^

Factor	Model 1: baseline		Model 2: cry for help		Model 3: fame seeking	
	OR (95% CI)	*P *value	OR (95% CI)	*P *value	OR (95% CI)	*P *value
Shooter aged ≤20 y	13.3 (2.8-63.7)	.001	4.4 (0.8-25.0)	.09	11.9 (2.3-61.4)	.003
Shooter identified as race/ethnicity other than White	0.4 (0.2-0.9)	.02	0.4 (0.2-1.2)	.10	0.4 (0.2-1.0)	.06
Location						
School or college	4.5 (1.1-17.7)	.03	4.6 (0.8-26.4)	.08	2.4 (0.5-13.0)	.30
Workplace	3.0 (1.2-7.6)	.02	3.5 (1.2-10.2)	.02	2.8 (1.1-7.6)	.04
Region						
South	1.8 (0.6-6.0)	.32	1.9 (0.5-7.5)	.36	1.2 (0.3-4.2)	.79
Northeast	0.2 (0.3-0.8)	.03	0.1 (0.0-0.9)	.04	0.1 (0.0-0.6)	.009
West	0.5 (0.1-1.8)	.30	0.4 (0.1-1.9)	.27	0.4 (0.1-1.5)	.16
Suicidality						
Suicidal before	NA	NA	3.7 (1.0-13.6)	.047	NA	NA
Intended to die	NA	NA	1.5 (0.5-4.9)	.48	NA	NA
Depressed mood	NA	NA	1.2 (0.4-3.1)	.74	NA	NA
Prior counseling	NA	NA	7.0 (2.0-24.8)	.003	NA	NA
Psychosis	NA	NA	0.3 (0.1-0.8)	.02	NA	NA
Psychiatric medication	NA	NA	1.0 (0.3-4.1)	.96	1.7 (0.4-7.6)	.46
Social media use	NA	NA	NA	NA	3.1 (0.9-11.0)	.08
Planning	NA	NA	NA	NA	1.3 (0.2-8.3)	.77
Performance	NA	NA	NA	NA	2.0 (0.5-7.6)	.29
Interest in past mass violence	NA	NA	NA	NA	0.3 (0.0-2.4)	.25
Fame-seeking motive	NA	NA	NA	NA	11.9 (2.3-61.4)	.003

Abbreviations: NA, not applicable; OR, odds ratio.

^a^ All models control for year the shooting took place.

#### Model 1

Model 1 includes a baseline logistic regression model for estimating leakage. This baseline model shows the tendency for young shooters to leak (odds ratio [OR], 13.3; 95% CI, 2.8-63.7). Additionally, shooters of a race or ethnicity other than White had lower odds of leaking their plans than White shooters (OR, 0.4; 95% CI, 0.2-0.9), while workplace shooters had higher odds of leaking their plans than shooters in other locations (OR, 3.0; 95% CI, 1.2-7.6).

#### Model 2

The variables of primary interest are included in models 2 (cry for help) and 3 (fame seeking). When several mental health factors were included in model 2, the association between age and leakage was reduced to nonsignificance, while perpetrators who attended counseling previously had 7 times the odds of leaking their plans than perpetrators who did not attend counseling previously (OR, 7.0; 95% CI, 2.0-24.8). There was a significant association between previous suicidality and leakage (OR, 3.7; 95% CI, 1.0-13.6). Perpetrators motivated by psychosis, on the other hand, had lower odds of leaking their plans than perpetrators not motivated by psychosis (OR, 0.3; 95% CI, 0.1-0.8).

#### Model 3

The fame-seeking model presented in model 3 did not affect the association of age with leakage from the baseline model. There were no associations between fame-seeking factors and the likelihood of leaking plans ahead of the shooting.

## Discussion

Consistent with previous studies of leakage among mass shooters,^15^ this study found that leakage was common among public mass shooters overall and particularly among school shooters and young shooters. However, this study asked the critical question of whether leakage was better described as a cry for help or fame-seeking behavior. The results show that leakage was most associated with the cry-for-help model, particularly for youth and those with serious mental health concerns. There was an association between leakage and suicidality and leakage and prior counseling, but at the same time, leakage was uncommon for perpetrators motivated by psychosis.

These findings have public health implications. Leakage was often nonspecific among perpetrators, meaning traditional threat assessment models focused on specific threats of violence or distinguishing transient from substantive threats may be too focused, missing critical opportunities for intervention.^24^ The finding that young shooters most often leaked to their peers further implies a need to educate young people about the dynamics of leakage and empower them to report leakage to an appropriate source when it occurs.

There is evidence that anonymous reporting systems increase reporting behavior in schools.^25^ Strong, trusting relationships between students and adults at school may also break down institutional codes of silence and increase the likelihood of reporting. If leakage is a cry for help, then we must ensure help is not only available but accessible and affordable. An inappropriate response to leakage will only deter it in the future, when leakage is in fact a critical moment for intervention that could avert a mass shooting and save lives.

### Limitations

This study has limitations. The data on public mass shootings (≥4 people killed in a public location) are not representative of all mass shootings, broadly defined, in the United States, and the limited number of cases in the study reduces the statistical power of the analyses. The database was built using publicly available information, which was necessary but leaves room for bias and misinformation because the source data were originally gathered for purposes different from our own.^26^ Some cases are well reported on, while others are not.^27^ Mass shootings in kindergarten through 12th grade schools and military bases, mass shootings with higher body counts or younger victims, shootings perpetrated with assault rifles, and shootings clustered with other shootings tend to receive more attention.^28,29^ We tend to know more about recent cases, which reflects better reporting over time and more advocacy and awareness around the topic of mass shootings. It is possible that perpetrators leaked their plans but that information was not made publicly available. It is also possible that perpetrators had an unknown mental health history that was not publicly available. Ideally, we would use medical records for diagnostic coding; however, the Health Insurance Portability and Accountability Act of 1996 and other data privacy laws limit full access to official records for validation purposes. This study used dichotomous variables to describe complex factors, such as social media usage and mental health histories. Seeking mental health services could be unrelated to a cry-for-help motive, and social media use could be unrelated to a fame-seeking motive. Future research could examine the motivations of people who leak their plans or threaten a shooting in more depth, using interviews or case studies.

## Conclusions

In this study, leakage was associated with a history of counseling and suicidality, suggesting that it could best be characterized as a cry for help from perpetrators prior to their act. Often, our institutional response to leakage is to punish the leaker with exclusionary practices like suspension, expulsion, or even criminal charges.^20^ However, if perpetrators who leak tend to be young and experiencing suicidal ideation, punitive measures could exacerbate their grievance and suicidality. Crisis response teams that respond to leakage with appropriate and holistic interventions, such as suicide prevention services, mental health services, or peer support, may more effectively prevent future violence.^23^
