# Errors in Table

**DOI:** 10.1001/jamanetworkopen.2022.16011

**Published:** 2022-05-13

In the Original Investigation titled “Communication of Intent to Do Harm Preceding Mass Public Shootings in the United States, 1966 to 2019,”^1^ published November 4, 2021, there were errors in Table 3. The odds ratios that appear in model 3 for psychiatric medication, social media use, planning, performance, interest in past mass violence, and fame-seeking motive should have read NA, 1.7 (0.4-7.6), 3.1 (0.9-11.0), 1.3 (0.2-8.3), 2.0 (0.5-7.6), and 0.3 (0.0-2.4), respectively. The *P *values should have read NA, .46, .08, .77, .29. and .25, respectively. This article has been corrected.^1^

## References

[1] Peterson J, Erickson G, Knapp K, Densley J. Communication of intent to do harm preceding mass public shootings in the United States, 1966 to 2019. JAMA Netw Open. 2021;4(11):e2133073. doi:10.1001/jamanetworkopen.2021.33073 34735012 PMC8569489

